# Venous stasis retinopathy in a ten-year-old boy with ocular hypertension: a case report

**DOI:** 10.1186/s12886-020-01662-z

**Published:** 2020-10-27

**Authors:** Julia V. Stingl, Laura Ponce Nunez, Alexander K. Schuster, Esther M. Hoffmann

**Affiliations:** Department of Ophthalmology, University Medical Center Mainz, Langenbeckstraße 1, 55131 Mainz, Germany

**Keywords:** Venous stasis retinopathy, Central retinal vein occlusion, Ocular hypertension, Valsalva maneuver, Case report

## Abstract

**Background:**

Central retinal vein occlusion is a variable disease pattern. Preliminary stages of a complete occlusion of the central vein, wich are subsumed under the term venous stasis retinopathy, may occur as transient blurred vision and with subtle alterations of the fundus. Course and prognosis are benign, visual acuity usually recovers. By now, venous stasis retinopathy in children due to Valsalva maneuver has not been described in literature yet.

**Case presentation:**

We present an impressive case of venous stasis retinopathy in a 10-year-old boy with ocular hypertension and megalocornea due to increased intraocular pressure provoked by Valsalva maneuver. Main symptom was transient blurred vision in the left eye. The intraocular pressure was 28 mmHg, fundus exam revealed tortuous veins and a flame shaped hemorrhage at 7 o’clock. Total recovery under topical antiglaucomatous therapy could be observed after 1 month.

**Conclusions:**

Acute increase in intraocular pressure, provoked by Valsalva maneuver is a risk factor for venous stasis retinopathy. Further general and vascular risk factors should be ruled out by extensive examination. Children with ocular hypertension might be at higher risk for impending vein occlusion as shown in this case.

## Background

Central retinal vein occlusion (CRVO) occurs with a prevalence of about 0,08% [1]. It is known to be a variable disease pattern with symptoms and findings ranging from transient blurred vision with tortuous veins to non-ischemic hemorrhagic retinopathy, a significant reduction in visual acuity due to macular edema and finally ischemic retinopathy with neovascularization and permanent visual loss. In 1976 S. S. Hayreh postulated the necessity to distinguish the full picture of CRVO with hemorrhagic retinopathy from its preliminary stage, the venous stasis retinopathy (VSR) [2]. We present an impressive case of venous stasis retinopathy in a 10-year-old boy due to increased intraocular pressure (IOP) provoked by Valsalva maneuver after being upset, wich has not been discribed as yet.

## Case presentation

A 10-year-old boy with a history of ocular hypertension, megalocornea and hypermetropia presented to our emergency department after referring transient blurred vision and subjectively impaired visual fields in his left eye accompanied by headache and eye ache the day before. At the onset of symptoms IOP was 28 mmHg in the left eye, measured by his general ophthalmologist. The patient’s father reported that during a dispute at home the boy got upset, agitated and tensed the evening before the symptoms presented. His face turned red and he stopped breathing for 10 sec in the sense of a Valsalva maneuver. There was no history of breath holding spells, but, according to the father, phases like this would already have happened before. At time of examination in our clinic (24 h later) regression of symptoms had occurred.

**Fig. 1:**
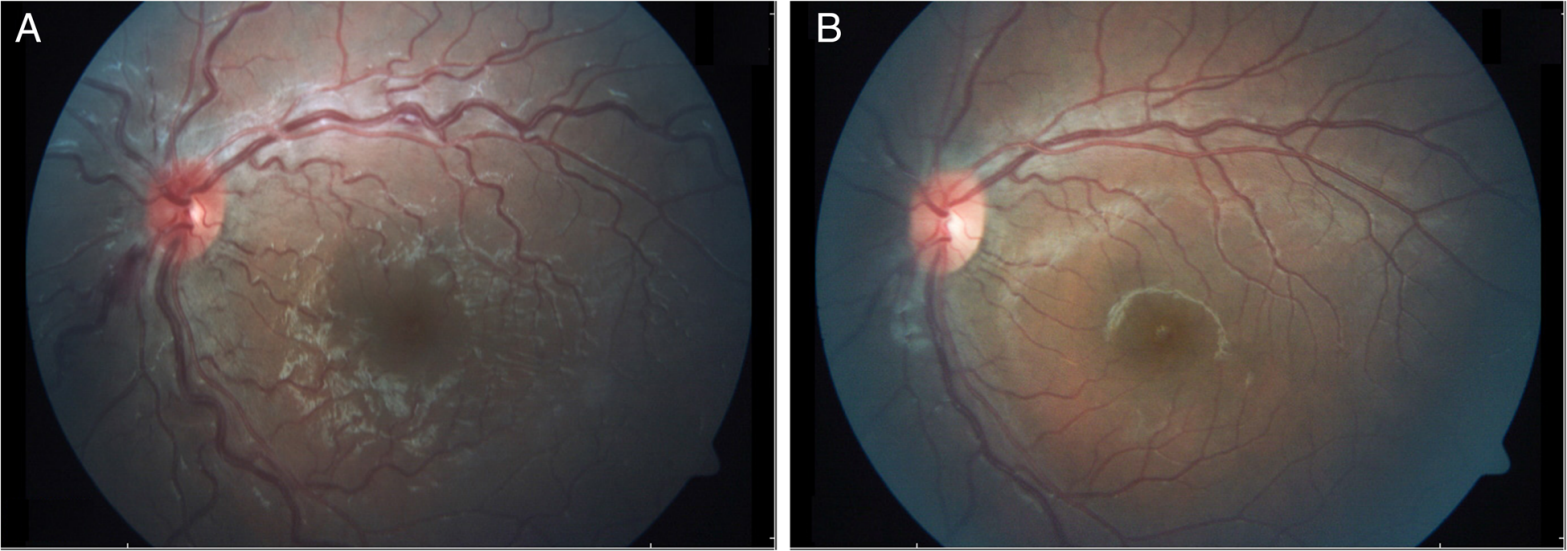
Fundus photography of the left eye. **a**, at the day of first examination fundus showed engorged and tortuos retinal veins, optic disc hyperemia and a flame shaped hemorrhage at the disc (at 7 o’clock). **b**, fundus photography 4 weeks later at follow-up with normal fundus findings

**Fig. 2:**
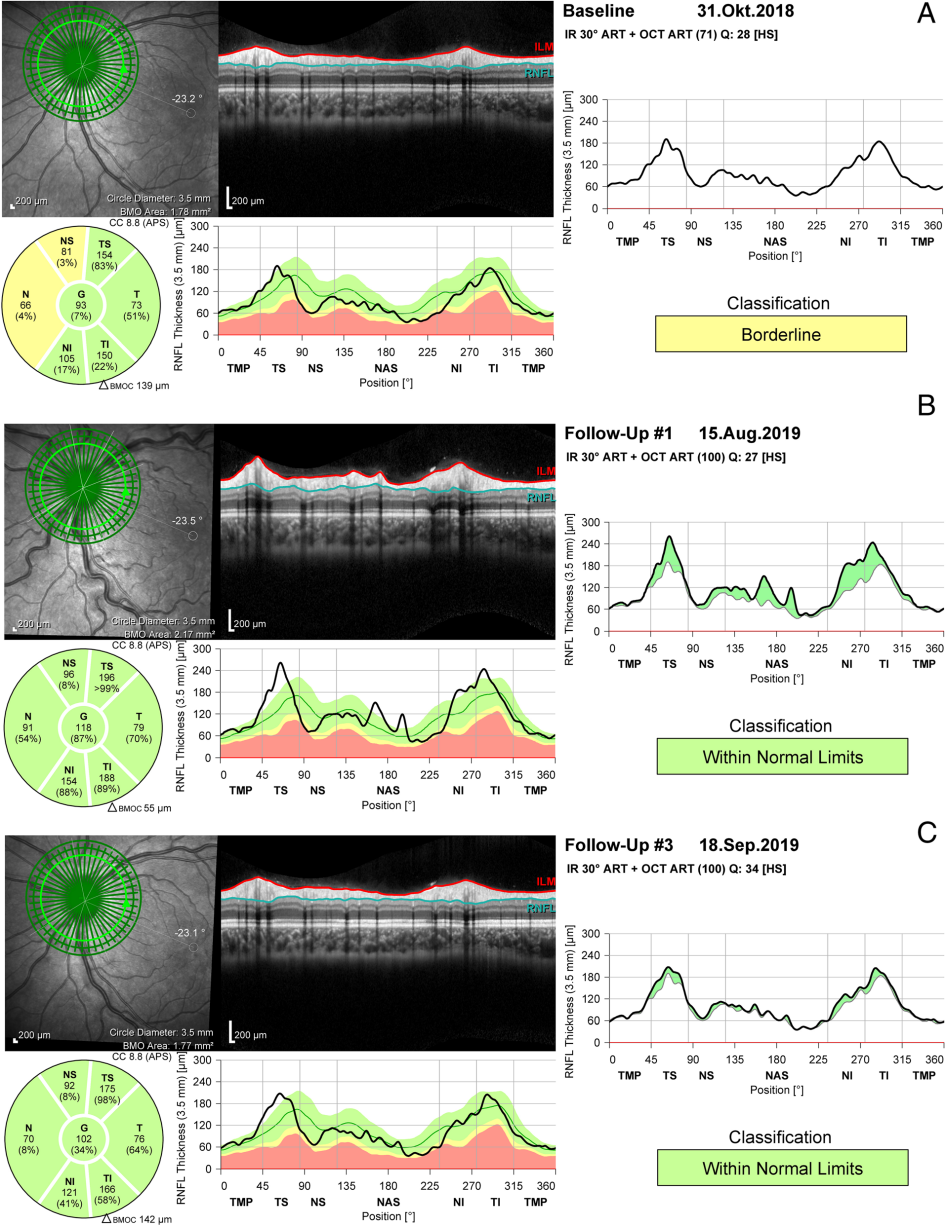
OCT of the left eye. **a**, RNFL thickness at baseline 1 year before first presentation of VSR symptoms. **b**, RNFL thickness at admittance due to VSR. The gently swoolen optic disc presents in RNFL thickness increase (black graph) compared to baseline RNFL thickness (grey graph) and reference database (green graph). Infrared photography shows tortuous retinal veins. **c**, RNFL thickness 1 month after first presentation of VSR symptoms. RNFL thickness decrease and regression of venous tortuosity have occured

At the time of admittance, best corrected visual acuity was 20/20 in the right eye and 20/25 in the left eye. IOP measured by Goldmann applanation tonometry was 16 and 12 mmHg, respectively. A relative afferent pupillary defect (RAPD) was absent. Slitlamp examination showed clear megalocorneae (white-to-white corneal diameter OD/OS 15.7/15.9 mm in biometric analysis) and normal anterior segment. Dilated fundus exam revealed tilted and hyperemic, minimally swollen optic disc with a flame shaped hemorrhage at 7 o’clock, markedly engorged and tortuous veins and one cotton-wool-like spot at the superior arcade (Fig. 1a). Octopus perimetry showed unspecified threshold elevations (OD/OS mean deviation 3.0/4.6 dB). Optical coherence tomography (OCT) of the optic disc showed a slight increase of retinal nerve fiber layer (RNFL) particularly left, compared to prior examinations (Fig. 2a and b).

**Fig. 3:**
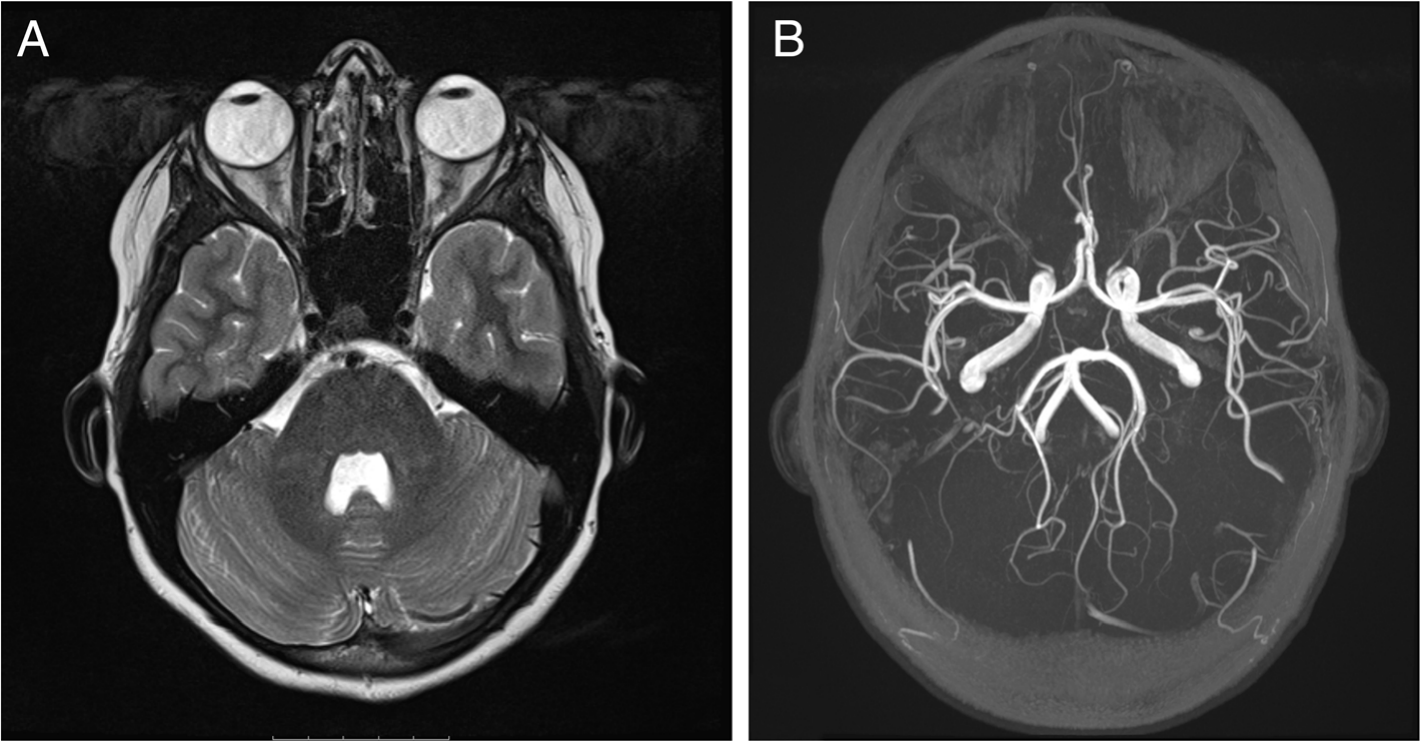
Magnetic resonance imaging and angiography. **a**, T2 weighted transverse image of the neurocranium. Note the slightly enlarged optic nerve sheds and tortuous optic nerves. Normal brain parenchyma, no signs for idiopathic intracranial hypertension or optic neuritis. **b**, Three-dimensional image of the time of flight angiography (TOF) showing normal intracranial vessels

During the hospital stay a monitoring of IOP over 72 h (also using Goldmann applanation tonometry) without any topical medication was carried out, and IOP values up to 34 mmHg in the right and 14 mmHg in the left eye were found. Under topical medication with Tafluprost once per day, the IOP decreased to values from 8 to 14 mmHg. A slightly increased blood pressure (up to 130/100 mmHg) was detected. A complete laboratory examination was performed. Neither full blood count nor electrolytes, infection, kidney or coagulation parameters, thrombophilia screening or autoantibodies were abnormal. Immunofixation showed a slight increase of alpha 2-globulines and a slight decrease of gamma-globulins. Borreliosis serology and virology (including cytomegaly, Herpes simplex, Varicella zoster and HIV) did not show any active infection. Magnetic resonance angiography (Fig. 3a and b) was performed to rule out any intracranial vessel anomaly. Besides normal cervical and intracranial vessels it showed slightly enlarged optic nerve sheds on both sides and an acute sinusitis on the right side. No optic neuritis was noted. Duplex sonography of the carotid arteries was normal.

A clinical diagnosis of VSR was made. The increased IOP and possibly the Valsalva maneuver were considered to be at least partly responsible for a transient venous thrombosis leading to venous stasis by applying pressure on the retinal veins in the prelaminar region of the optic disc. A cause for an optic neuritis could not be found in the laboratory and imaging diagnostics.

The patient was treated with Tafluprost eye drops once a day. At follow-up 4 weeks later, the fundus showed a decrease in venous tortuosity, the optic disc hemorrhage was reabsorbed (Fig. 1b). VA was OD 20/15 and OS 20/20. IOP was 22 mmHg on both sides, the Tafluprost eye drops had not been administered the evening before the follow-up examination. Octopus perimetry revealed a slight mean deviation improvement of − 1.2 dB in the left eye. Optic disc optical coherence tomography (OCT) showed a decrease in retinal nerve fiber layer (RNFL) (Fig. 2c). Blurred vision or obscurations had not occurred since hospital discharge.

## Discussion and conclusions

We present a case of VSR after Valsalva maneuver in a 10-year-old boy. To the authors’ knowledge neither VSR at this age nor VSR after Valsalva maneuver have been reported in literature yet.

VSR was first described by Hayreh as an incomplete occlusion of the central retinal vein [3]. Main symptom is a blurred, oftenly intermittent central vision that lasts only few seconds to hours. The complaints may be worse in the morning and improve throughout the day because in a lying position the central venous pressure is higher than in an upright position, which decreases the venous outflow and enhances the venous stasis. Visual acuity is clearly better as usually seen with complete occlusion of the central vein. Fundoscopy shows tortuous and engorged retinal veins and frequently spot or flame shaped retinal hemorrhages as well as hyperemic and gently swollen optic discs. Cotton-wool spots are rather uncommon but possible. Macular edema may be present.

In fluorescein retinal angiography a venous stasis with optionally dilatated capillaries and microaneurysms may be observed. The main veins show fluorescein leakage especially in the concavities. Obliterated capillaries or neovascularizations are typically absent [3]. Fundus autofluorescence may show a fern like perivenular appearance [4, 5].

Hayreh gathered from his observations that VSR occurs more frequently in older adults than in younger [3]. To sum up pathogenesis of CRVO we can rely on the Virchow’s Triad which describes the causes of thrombosis:

1. *Damage of the vessel wall*, e.g. by inflammation or trauma. Hayreh postulated a so-called optic disc vasculitis to be etiologic in young VSR patients [6, 7].
2. *Altered blood flow*. A pathological compression of the central retinal vein due to arteriosclerosis in the central artery is described to be the main cause of VSR in older people [3]. Also a swollen optic disc and a high IOP can cause a narrowing of the central vein which results in stasis and activation of the plasmatic coagulation [2, 8]. An impaired circulation due to low arterial pressure and high central venous pressure in lying position during the night furthermore explains the blurred vision in the morning [8], and worsening of the blurred vision after Valsalva, stooping or orthostatic hypotension have been reported in the literature [7].
3. *Alteration of the blood composition*, such as increased blood viscosity or thrombophilia [8].

Risk factors for CRVO are arterial hypertension, diabetes mellitus, glaucoma, elevated IOP and carotid artery plaques [9, 10]. According to “The Eye Disease Case-Control Study Group” open-angle glaucoma and high IOP are the most important risk factors seen in CRVO patients [9].

VSR usually is self-limited and has a good outcome without any treatment [2, 11]. Therapeutic attempts with glucocorticoids or IOP lowering eye drops have been made, but there is no evidence of efficacy of any treatment in eyes without macular edema, ischemia or neovascularization [3, 5, 12]. Limiting factor of the visual acuity is the presence of a macular edema which can lead to macular degeneration and should be treated with anti-vascular endothelial growth factor (anti-VEGF) therapy [3, 13]. The risk of progression to a full obstruction of the central vein has been mentioned in the literature but not proved [11].

The term Valsalva maneuver describes the pressing of exhaled air against the closed glottis, which leads to a risen intrathoracic and intraabdominal pressure. This reduces the venous backflow to the heart and subsequently increases the pressure in the peripheral veins. Valsalva maneuver can cause a rupture of perifoveal capillaries with retinal hemorrhage [14, 15] and rise the IOP [16, 17]. The reduced venous backflow leads to a congestion of the retinal veins, which on the one hand may result in a venous stasis triggering a thrombosis, and on the other hand may produce a localized edema that - in the area of the optic disc and the lamina cribrosa - enhances the reduced venous reflux. Additionally, the rise of the intraocular pressure leads to compression and collapsing of the retinal veins, which also may induce a central retinal vein occlusion or its preliminary stages [2, 18].

Limitation of this report is its single nature, since we cannot present a case series. However, this might be a strength of the paper: By emphasizing the unique feature of our patient we point out typical symptoms of VSR which might be underdiagnosed and more common than expected. Therefore, this case contributes to the ophthalmological education and sensitizes to this disease pattern.

Acute increase in IOP, provoked by Valsalva maneuver is a risk factor for VSR. Further general and vascular risk factors should be ruled out by extensive examination. Children with ocular hypertension might be at higher risk for impending vein occlusion as shown in this case.

## Data Availability

Data sharing is not applicable to this article as no datasets were generated or analysed during the current study.

## Abbreviations

CRVO: Central retinal vein occlusion
VSR: Venous stasis retinopathy
IOP: Intraocular pressure
OCT: Optical coherence tomography
RNFL: Retinal nerve fiber layer
